# Comparison of magnetic resonance feature tracking for longitudinal strain calculation with spatial modulation of magnetization imaging analysis

**DOI:** 10.1186/1532-429X-15-S1-P123

**Published:** 2013-01-30

**Authors:** William E Moody, Robin J Taylor, Nicola C Edwards, Fraz Umar, Colin D Chue, Tiffany J Taylor, Charles J Ferro, Jonathan N Townend, Francisco Leyva, Richard Steeds

**Affiliations:** 1 Cardiology, University of Birmingham and Queen Elizabeth Hospital Birmingham, Birmingham, UK

## Background

Feature-tracking (FT) analysis offers a novel, fast and practicable method to calculate strain from routinely acquired steady state free precession (SSFP) images without the need to perform additional tagged sequences. There is no validation of this technique, however, against a reference standard myocardial tagging analysis for any strain parameter other than mid-left ventricular whole slice circumferential strain in children. In an adult study of patients with dilated cardiomyopathy (DCM) and healthy controls, we sought to validate the FT method (TomTec Imaging systems, Munich) against spatial modulation of magnetization (SPAMM) tissue tagging analysis (Cardiac Image Modeling Package (CIMTAG), University of Auckland) for the computation of long axis function.

## Methods

We compared measures of peak systolic longitudinal strain from the horizontal long axis view using the 2 techniques in 30 patients (median age 42 yr, male 40%). Normal healthy adults were identified from an ongoing prospective, observational research study examining the effects of living kidney donation on cardiovascular structure and function (NCT01028703) while DCM patients underwent routine tagging during CMR studies performed for clinical requirements. A timed retrospective off-line analysis was performed on matched tagged and SSFP slices by 2 independent blinded observers (WEM and RJT).

## Results

**Figure 1:**
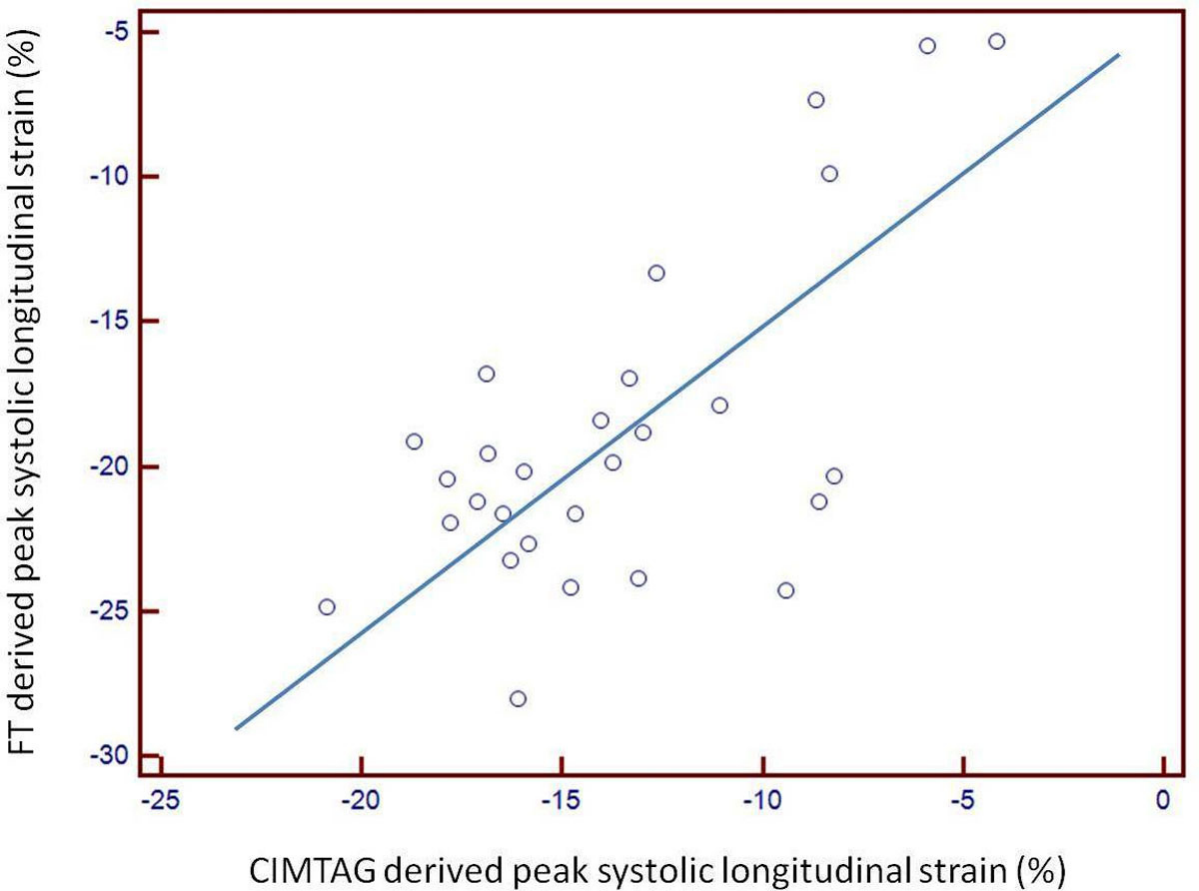
In an analysis of all patients, mean FT systolic longitudinal strain values were highly correlated with CIMTAG values, with a Pearson correlation coefficient of 0.67 (P=0.0001).

**Figure 2:**
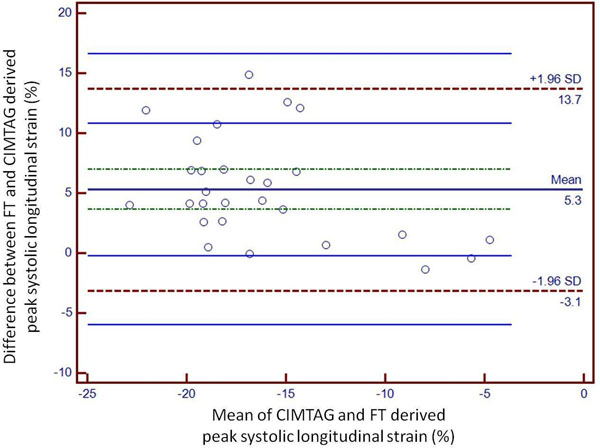
Bland-Altman plot showing consistent overestimation of FT derived peak systolic longitudinal strain values compared with the CIMTAG reference standard.

Mean peak systolic longitudinal strain and strain rate determined by FT were significantly reduced in DCM patients compared with healthy controls (-6.7 ± 1.7% vs. -20.4 ± 3.7%, P<0.001 and -1.21 ± 0.14 /s vs. -0.61 ± 0.09 /s, both P<0.0001). The mean peak systolic longitudinal strain of DCM patients determined by CIMTAG (-6.7 ± 1.7%) and FT (-7.0 ± 1.4%) was not significantly different (P = NS). In an analysis of all patients, mean FT systolic longitudinal strain values were highly correlated with CIMTAG values, with a Pearson correlation coefficient of 0.67 (P=0.0001, Figure 1). The individual longitudinal systolic strain values obtained for each subject were also compared by the Bland-Altman technique which showed that FT software consistently overestimated CIMTAG derived longitudinal strain values (Figure 2). Intraobserver and interobserver variability for FT analysis was low (0.45 ± 0.38% and 0.66 ± 1.2%, respectively). The average time taken for post-processing strain analysis using FT software was significantly less than that required for CIMTAG (5.2 ± 3.1min vs. 25.1 ± 4.2 min, P<0.0001).

## Conclusions

This FT based assessment of longitudinal strain correlated highly with values derived from tagged images in a population with a wide range of left ventricular function. Furthermore, FT can be performed without the need for additional imaging and lengthy post-processing.

## Funding

WEM is funded by a BHF Clinical Research Fellowship

